# *Helicobacter pylori* infection is independently associated with triglyceride levels: a propensity score–matched cross-sectional study

**DOI:** 10.3389/fendo.2026.1792530

**Published:** 2026-03-25

**Authors:** Yu Zhou, Jie Xu, Yating Shi, Ge Yu, Hui Duan, Zhaoyi Chen, Daoxing He

**Affiliations:** 1 Department of Gastroenterology, Xuancheng People’s Hospital, Affiliated Xuancheng Hospital Wannan Medical College, Xuancheng, Anhui, China; 2 Huai’an No.3 People’s Hospital, Huai’an, Jiangsu, China; 3 Department of Gastroenterology, Shanghai General Hospital, Shanghai Jiaotong University School of Medicine, Shanghai, China

**Keywords:** dyslipidemia, helicobacter pylori, paired fixed-effects regression, propensity score matching, triglyceride

## Abstract

**Background:**

*Helicobacter pylori* (*H. pylori*) infects more than 50% of the global population and is associated with a variety of upper gastrointestinal diseases. In recent years, studies have suggested that *H. pylori* infection may contribute to metabolic syndrome and dyslipidemia.

**Objective:**

To investigate the association between *H. pylori* infection and serum lipid levels.

**Methods:**

We enrolled 678 participants who underwent both the ^13^C-urea breath test and a lipid profile test at our hospital in 2024. We collected demographic and clinical characteristics, breath test results, and serum lipid profiles. *H. pylori* positivity was defined as a DOB value ≥ 4‰. We performed 1:1 propensity score matching (PSM) on key covariates (sex, age, and body mass index [BMI]), yielding 214 *H. pylori*-positive participants and 214 *H. pylori*-negative participants. We compared serum lipid levels between the two matched groups. We used paired fixed-effects linear regression to evaluate the independent association between *H. pylori* infection and triglyceride (TG) levels.

**Results:**

Triglyceride (TG) levels remained higher in the *H. pylori*-positive group. Total cholesterol (TC), low-density lipoprotein cholesterol (LDL-C), and high-density lipoprotein cholesterol (HDL-C) did not differ significantly between the two groups. In the adjusted model controlling for age, sex, smoking, alcohol use, hypertension, and diabetes, *H. pylori* infection was independently associated with higher TG levels.

**Conclusion:**

*H. pylori* infection is independently associated with elevated triglyceride levels. These findings highlight the potential role of *H. pylori* in metabolic risk assessment and in the design of intervention studies. The causal relationship requires further confirmation in prospective and mechanistic studies.

## 1 Introduction

Dyslipidemia, particularly elevated triglycerides (TG), is a major risk factor for atherosclerotic cardiovascular disease and other metabolism-related disorders. Its development is closely linked to insulin resistance, chronic inflammation, and gut microbiota dysbiosis. With lifestyle changes in China, the prevalence of dyslipidemia has continued to rise, and hypertriglyceridemia has received increasing attention in the primary prevention of cardiovascular disease (1). Beyond traditional genetic and lifestyle determinants, identifying new and modifiable risk factors for hypertriglyceridemia is essential to improve risk stratification and optimize preventive and therapeutic strategies.

*Helicobacter pylori* (*H. pylori*) is among the most common chronic bacterial infections and is strongly associated with chronic gastritis, peptic ulcer disease, gastric cancer, and other gastrointestinal disorders (2, 3). Growing evidence suggests that *H. pylori* infection may disrupt metabolic homeostasis through systemic low-grade inflammation (3), altered gastrointestinal hormone secretion (4), and changes in the gut microbiota (5).

Since the 1990s, multiple studies have reported associations between *H. pylori* infection and higher total cholesterol (TC), TG, and low-density lipoprotein cholesterol (LDL-C), potentially increasing the risk of coronary heart disease (6–9). A prospective cohort study by Xie et al. reported that *H. pylori* infection was independently associated with higher TG levels among Chinese women (10).However, findings across studies remain inconsistent (11–13). Therefore, using *H. pylori* status determined by the ^13^C-urea breath test, we constructed matched pairs via propensity score matching and evaluated the association between *H. pylori* infection and TG levels within a paired fixed-effects framework.

## 2 Methods

### 2.1 Subjects

**Figure 1:**
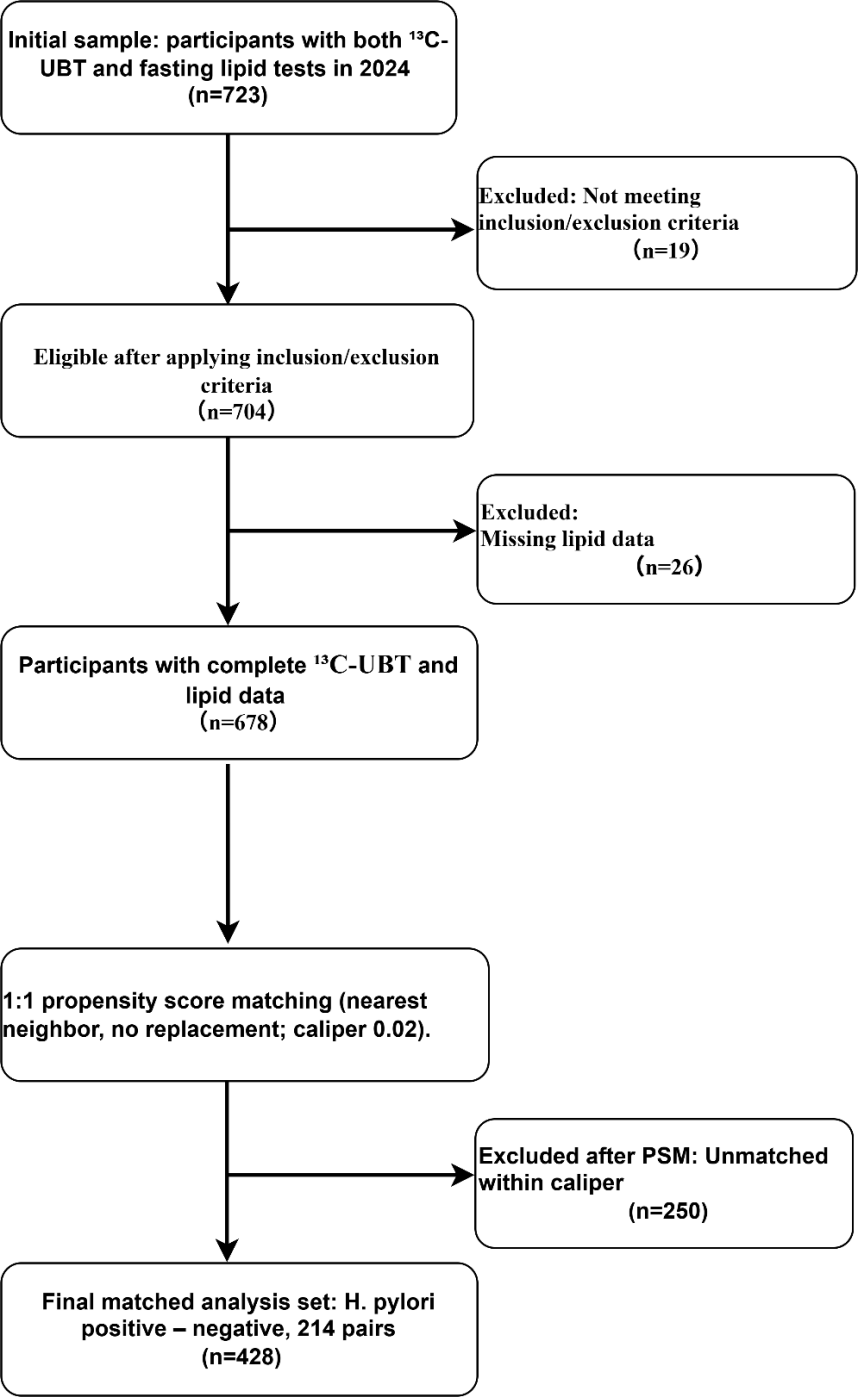
Flowchart of participant selection and propensity score matching.

This single-center cross-sectional study enrolled 723 adults who underwent both the ^13^C-urea breath test and serum lipid testing at our hospital in 2024. After applying prespecified inclusion and exclusion criteria, 19 participants were excluded, and an additional 26 were excluded because of missing key information, leaving 678 participants for analysis. We then performed 1:1 propensity score matching based on sex, age, and body mass index (BMI), resulting in 214 matched pairs (428 participants) for the primary analysis (**Figure 1**). Inclusion criteria were: (a) age ≥ 18 years; and (b) availability of key variables. Exclusion criteria were: (a) previously diagnosed dyslipidemia or long-term lipid-lowering therapy; (b) thyroid dysfunction, nephrotic syndrome, or malignancy; (c) long-term use of medications that may substantially affect lipid metabolism (e.g., statins or fibrates); and (d) incomplete data or missing key variables.

### 2.2 Data collection and measurements

#### 2.2.1 General clinical data

Sex, age, height, weight, smoking and alcohol consumption status, and histories of hypertension and diabetes were extracted from the hospital information system. BMI was calculated as weight (kg) divided by height squared (m^2^). Smoking, alcohol use, hypertension, and diabetes were coded as dichotomous variables according to the presence or absence of a long-term history.

#### 2.2.2 Assessment of *H. pylori* infection

All participants were assessed for *H. pylori* infection using the ^13^C-urea breath test. After fasting for at least 2 h and resting, a baseline breath sample (0 min) was collected. Participants then ingested 75 mg ^13^C-urea orally, and a second breath sample was collected 30 min later. Both samples were analyzed by isotope ratio mass spectrometry to calculate the delta over baseline (DOB) value. According to the manufacturer-recommended cutoff, a DOB value ≥ 4‰ was defined as *H. pylori* positive, and a DOB value < 4‰ as *H. pylori* negative.

#### 2.2.3 Measurement of lipid indicators

Fasting venous blood samples were collected in the morning from all participants. Serum total cholesterol (TC) and triglycerides (TG) were measured using enzymatic assays (TC by the CHOD-PAP method; TG by the GPO-PAP method). Low-density lipoprotein cholesterol (LDL-C) was measured using the surfactant clearance method, and high-density lipoprotein cholesterol (HDL-C) using a selective inhibition method. Abnormal lipid values were defined according to our laboratory reference ranges: TC > 5.2 mmol/L, TG > 1.7 mmol/L, LDL-C > 3.1 mmol/L, and HDL-C < 1.1 mmol/L.

### 2.3 Statistical analysis

All analyses were conducted using SPSS 25.0 and Python. Tests were two-sided, with a significance level of α = 0.05. Continuous variables are presented as mean ± standard deviation or median (interquartile range), as appropriate. Categorical variables are presented as counts and percentages. Effect estimates are reported as β (mmol/L) with 95% confidence intervals (CIs), together with P values and the number of matched pairs.

#### 2.3.1 Propensity score matching and balance assessment

Propensity scores were estimated using logistic regression with *H. pylori* infection status as the dependent variable and sex, age, BMI, smoking, alcohol use, hypertension, and diabetes as covariates. We performed 1:1 nearest-neighbor matching without replacement using a caliper of 0.02 on the logit of the propensity score. Covariate balance after matching was assessed using standardized mean differences, with values < 0.10 indicating adequate balance. Baseline characteristics and lipid measures after matching are summarized in **Table 1**.

**Table 1:** Baseline characteristics before and after propensity score matching.

Variable	Before matching			After matching		
	H. pylori− (n=450)	H. pylori+ (n=228)	SMD	H. pylori− (n=214)	H. pylori+ (n=214)	SMD
Age (years)	55.28± 11.77	53.21± 12.08	-0.174	52.81 ± 11.42	53.35 ± 12.16	0.050
BMI (kg/m^2^)	23.14 ± 3.05	24.20 ± 3.70	0.313	23.81 ± 3.31	24.02 ± 3.55	0.061
Male (%)	199 (44.2%)	134 (58.8%)	0.291	119 (55.6%)	121 (56.5%)	0.019
Smoking (%)	85 (18.9%)	56 (24.6%)	0.138	50 (23.4%)	50 (23.4%)	0.000
Alcohol (%)	55 (12.2%)	43 (18.9%)	0.183	36 (16.8%)	38 (17.8%)	0.025
Hypertension (%)	117 (26.0%)	68 (29.8%)	0.085	64 (29.9%)	62 (29.0%)	-0.021
Diabetes (%)	37 (8.2%)	31 (13.6%)	0.172	24 (11.2%)	25 (11.7%)	0.015
TC (mmol/L)	4.71 ± 0.97	4.83 ± 0.95	0.125	4.73 ± 0.98	4.81 ± 0.95	0.083
TG (mmol/L)	1.41 ± 0.82	1.76 ± 1.24	0.333	1.50 ± 0.97	1.78 ± 1.27	0.248
HDL-C (mmol/L)	1.27 ± 0.33	1.21 ± 0.32	-0.185	1.25 ± 0.31	1.21 ± 0.32	-0.127
LDL-C (mmol/L)	2.61 ± 0.72	2.70 ± 0.71	0.126	2.68 ± 0.73	2.69 ± 0.70	0.014

Values are mean ± SD or n (%). SMD = standardized mean difference; balance considered acceptable when |SMD| < 0.10. Group labels: H. pylori− (negative) and H. pylori+ (positive).

#### 2.3.2 Comparisons between matched groups

**Figure 2:**
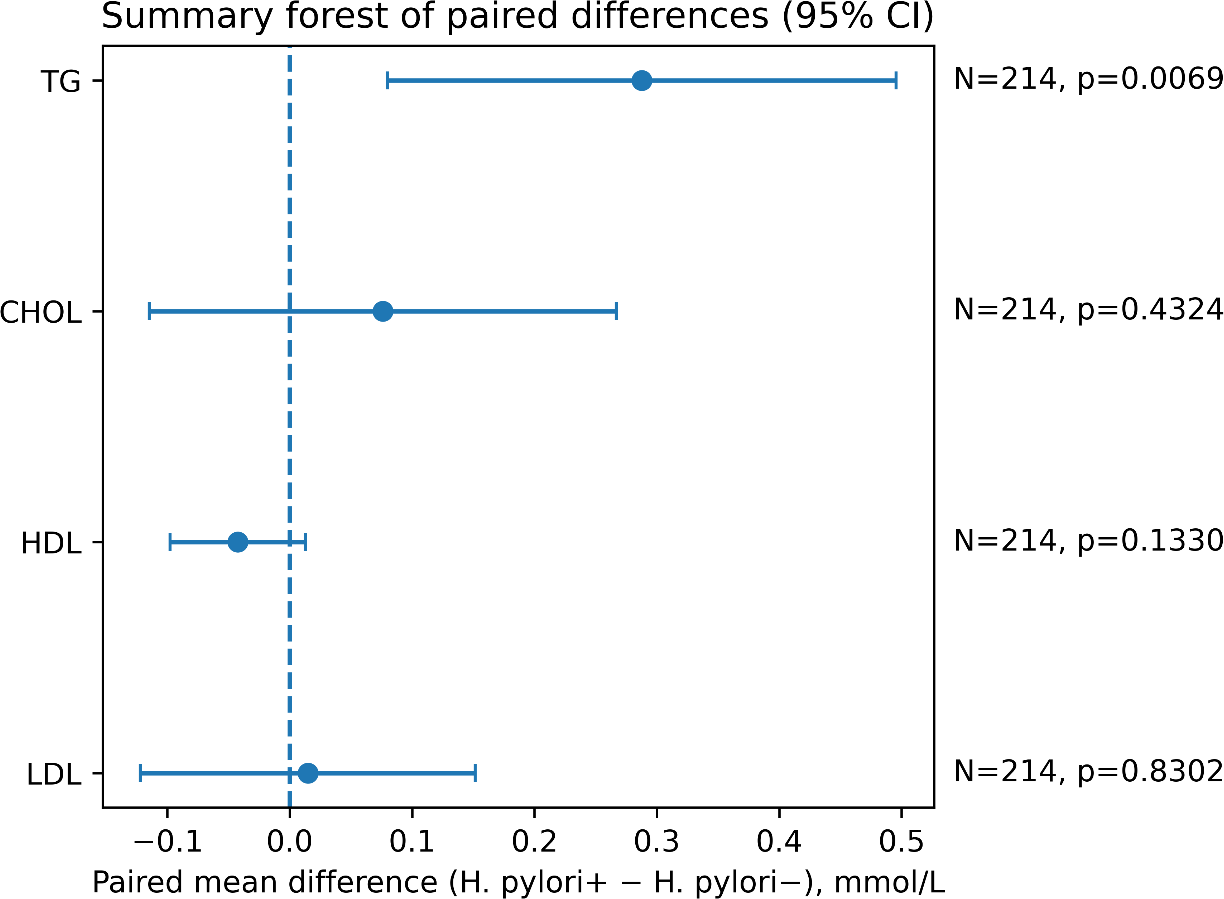
Summary Forest plot of lipid differences (CHOL, HDL, LDL, TG) after matching.

**Figure 3:**
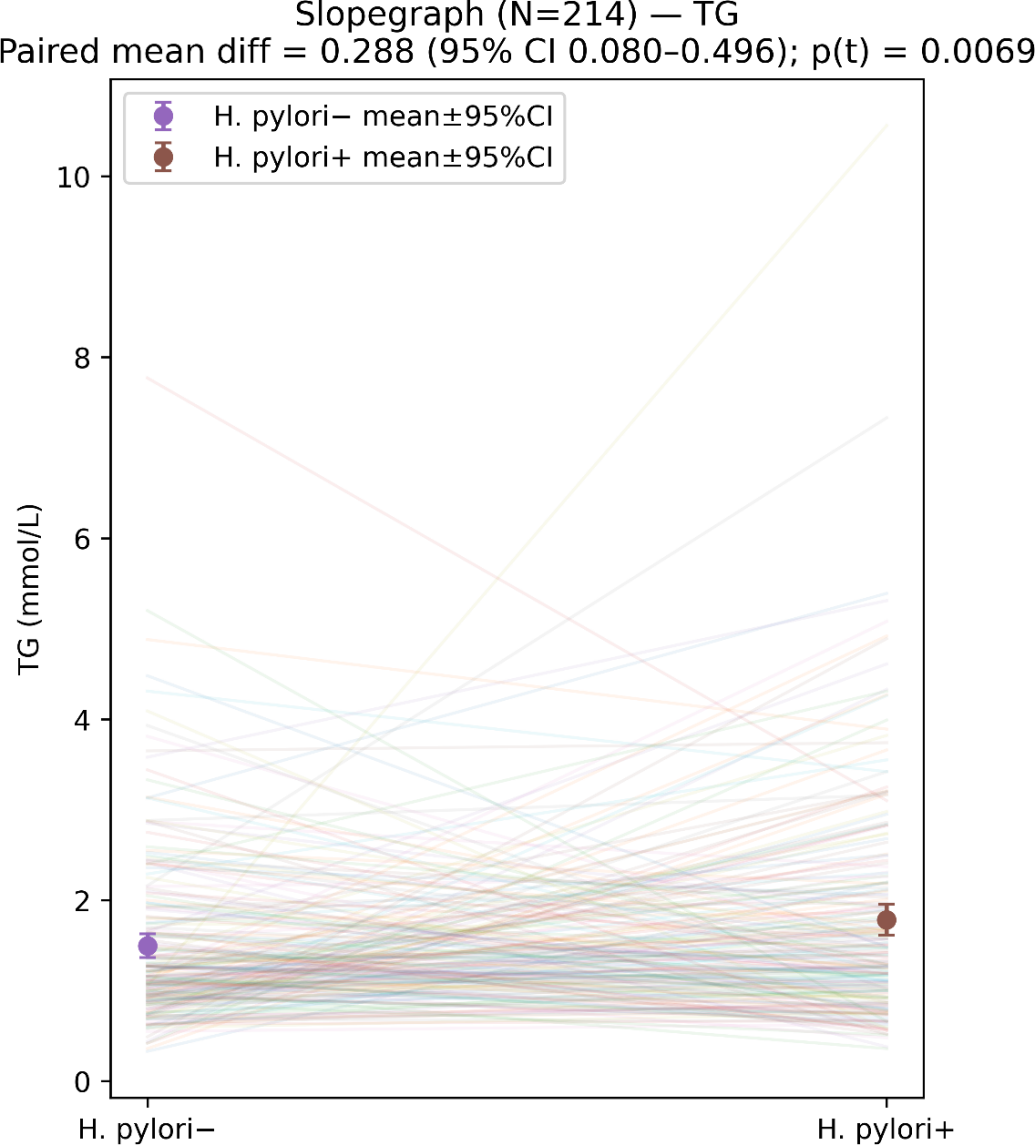
Within-pair TG change: H. pylori positive vs negative.

For paired categorical variables, the McNemar test (or paired χ^2^ test, as appropriate) was used. The distribution of continuous variables was assessed using the Kolmogorov–Smirnov test. Paired t-tests were applied for approximately normally distributed variables, and the Wilcoxon signed-rank test was used for non-normally distributed variables. Differences in the four lipid measures between matched groups are displayed in a summary forest plot (**Figure 2**), and within-pair changes in TG are illustrated using a paired connection plot (**Figure 3**).

#### 2.3.3 Paired fixed-effects regression models

In the matched sample, we fitted paired fixed-effects linear regression models to evaluate the association between *H. pylori* infection and TG levels, using cluster-robust standard errors at the matched-pair level. Covariates in this model were prespecified and retained based on prior literature and common clinically relevant factors. Four models were specified: Model 1 (M1) included *H. pylori* infection only; Model 2 (M2) additionally adjusted for sex, age, smoking, alcohol use, hypertension, and diabetes; Model 3 (M3) further adjusted for BMI; and Model 4 (M4) added HDL-C to Model 2. For each model, we report β coefficients, 95% CIs, P values, and the number of matched pairs.

## 3 Results

### 3.1 Baseline characteristics after propensity score matching

After 1:1 propensity score matching, 214 *H. pylori*-positive participants and 214 *H. pylori*-negative participants were included, yielding a total of 428 matched participants. Covariates including sex, age, body mass index (BMI), smoking, alcohol use, hypertension, and diabetes were well balanced between the two groups after matching, with absolute standardized mean differences for all covariates < 0.10 (**Table 1**).

### 3.2 Differences in serum lipid levels between matched groups

In the matched paired sample, differences in the four lipid measures are summarized in the forest plot (**Figure 2**). TG levels were higher in the *H. pylori*-positive group than in the *H. pylori*-negative group, and the difference was statistically significant. In contrast, there were no significant between-group differences in total cholesterol (TC), HDL-C, or LDL-C. Within-pair changes in TG are illustrated in the paired connection plot (**Figure 3**). In most matched pairs, TG tended to be higher when *H. pylori* were positive, which was consistent with the summary comparison.

### 3.3 Main analysis: paired fixed-effects linear regression

Within the paired fixed-effects framework, we fitted four stepwise models to evaluate the association between *H. pylori* infection and TG levels (**Table 2**). In Model 2 (M2), which adjusted for basic covariates, *H. pylori* positivity was independently associated with higher TG levels. After further adjustment for BMI in Model 3 (M3), the effect estimate was modestly attenuated compared with M2, suggesting that BMI may lie on the pathway linking *H. pylori* infection to TG levels. In the exploratory Model 4 (M4), which added HDL-C to M2, the association remained in the same direction.

**Table 2:** Effect of H. pylori on TG across models (within-pair FE; clustered SE).

Model	β (mmol/L)	95% CI	p	N pairs
M1	0.288	(0.081, 0.494)	0.006	214
M2	0.278	(0.077, 0.478)	0.007	214
M3	0.229	(0.021, 0.438)	0.031	214
M4	0.241	(0.048, 0.434)	0.014	214

Within-pair fixed-effects (demeaned) linear models with cluster-robust SEs by pair. M1: unadjusted; M2: analyzed adjusted for age, sex, smoking, alcohol, hypertension and diabetes; M3: M2 + BMI; M4: M2 + HDL (exploratory). β denotes mean difference in TG (mmol/L) for H. pylori+ vs H. pylori−.

### 3.4 Heterogeneity analysis

To assess effect modification by BMI, we added an *H. pylori* × BMI interaction term to the main model. The interaction was not statistically significant when BMI was analyzed as a dichotomous variable (≥ 24 vs. < 24) or as a continuous variable (all P > 0.05), suggesting that the association was broadly consistent across BMI levels; therefore, stratified results are not presented.

## 4 Discussion

This single-center study was conducted in a health check-up population. We applied 1:1 propensity score matching to balance major confounders, including age, sex, body mass index (BMI), smoking, alcohol use, hypertension, and diabetes. The matched pairs were then analyzed using a paired fixed-effects linear regression framework. We found that *H. pylori* infection was independently associated with higher triglyceride (TG) levels, whereas no consistent differences were observed for total cholesterol (TC), high-density lipoprotein cholesterol (HDL-C), or low-density lipoprotein cholesterol (LDL-C) after matching. After BMI was added to the model, the estimated association between *H. pylori* and TG was modestly attenuated, suggesting that BMI may lie on the pathway linking infection to TG. In an exploratory model that further adjusted for HDL-C, the direction of the association remained unchanged. Collectively, these findings support an association between *H. pylori* infection and elevated TG levels.

Our findings are generally consistent with previous observational studies. Early reports suggested that *H. pylori* infection was associated with higher TC, TG, and LDL-C levels and might increase the risk of coronary heart disease through adverse changes in lipid profiles (7, 8). Studies across multinational populations have further shown close associations between *H. pylori* infection and increased TC, decreased HDL-C, and dyslipidemia, particularly elevated TG (14–16). Clustering of *H. pylori* infection with atherosclerotic risk factors—such as increased TG and LDL-C and decreased HDL-C—has also been reported in Ethiopian and sub-Saharan African populations (17, 18). In Chinese populations, including diabetes cohorts and prospective follow-up studies, *H. pylori* infection was associated with a higher risk of dyslipidemia, especially increased TG (4, 10). In line with this literature, we observed an independent association between *H. pylori* infection and elevated TG, but not TC, HDL-C, or LDL-C, suggesting that different lipid components may respond differently to infection and that TG may be more sensitive to *H. pylori*–related metabolic perturbations (19).

Several biological mechanisms may explain this association. Chronic infection can induce systemic low-grade inflammation (1), enhance hepatic synthesis of very-low-density lipoprotein (VLDL), and inhibit lipoprotein lipase activity (5), thereby reducing TG clearance (20). Infection-related inflammation may also influence gastrointestinal hormone secretion (e.g., ghrelin and leptin), alter energy intake and fat distribution, and consequently affect lipid metabolism (2, 5). In addition, *H. pylori* infection has been linked to nonalcoholic fatty liver disease and metabolic syndrome; hepatic fat accumulation and insulin resistance can further promote hypertriglyceridemia (1, 5, 21). Evidence from intervention studies and meta-analyses also supports a potential benefit of eradication therapy: several studies reported reductions in TC and LDL-C or overall improvement in lipid profiles after eradication, accompanied by remodeling of the gut microbiota. These findings suggest that a gut microecology–inflammation–insulin resistance pathway may play a key role (5, 12, 22–24).

This study has several strengths. First, the matched design combined with paired fixed-effects modeling strengthened confounding control at both the design and analysis stages, particularly for unmeasured time-invariant factors within matched pairs. Second, the use of multiple stepwise models and consistency checks yielded stable conclusions in both direction and magnitude. Nevertheless, several limitations should be acknowledged. First, as a single-center retrospective study, the generalizability of the findings may be limited. Second, important lifestyle factors such as dietary patterns and physical activity were not available, nor were additional laboratory markers (e.g., white blood cell count, hemoglobin, and routine blood biochemistry), and residual confounding cannot be excluded. Third, we lacked longitudinal lipid data before and after eradication therapy; therefore, causal inference remains limited and should be evaluated in studies with stronger designs. Future multicenter prospective cohorts or randomized controlled trials incorporating eradication therapy, gut microbiota sequencing, and inflammatory markers are warranted to clarify the mechanisms underlying the *H. pylori*–microecology–metabolism axis in relation to TG regulation (25).

In conclusion, our study found an independent association between *H. pylori* infection and elevated TG levels, suggesting that *H. pylori* infection may be a potential risk factor for hypertriglyceridemia.

## 5 Conclusions

*H. pylori* infection was independently associated with elevated triglyceride levels, suggesting a potential role in metabolic risk assessment and the design of intervention strategies. However, the causal relationship and underlying mechanisms require further clarification through prospective studies and interventional trials.

## Data Availability

The raw data supporting the conclusions of this article will be made available by the authors, without undue reservation.
