# Supplementary material for: Helicobacter pylori infection is independently associated with triglyceride levels: a propensity score–matched cross-sectional study
Source: Front Endocrinol (Lausanne). 2026 Mar 25;17:1792530. doi: 10.3389/fendo.2026.1792530 (PMC13056812; doi:10.3389/fendo.2026.1792530)
Supplement: Supplementary file 1 [file Table1.docx]

Table S2 Comparison of lipid levels between the positive and negative groups of Helicobacter pylori before and after matching

| Lipid | **Before Matching** | | | **After matching** | | | | |
| --- | --- | --- | --- | --- | --- | --- | --- | --- |
|  | H. pylori−（n=450） | H. pylori+（n=228） | P value | H. pylori−（n=214） | H. pylori+（n=214） | Mean diff | 95%CI | P value |
| TC | 4.71 ± 0.97 | 4.83 ± 0.95 | 0.1234 | 4.73 ± 0.98 | 4.81 ± 0.95 | 0.08 | (-0.11, 0.27) | 0.4324 |
| TG | 1.41 ± 0.82 | 1.41 ± 0.82 | <0.001 | 1.50 ± 0.97 | 1.50 ± 0.97 | 0.29 | (0.08, 0.50) | 0.0069 |
| HDL-C | 1.27 ± 0.33 | 1.27 ± 0.33 | 0.0106 | 1.25 ± 0.31 | 1.21 ± 0.32 | -0.04 | (-0.10, 0.01) | 0.1330 |
| LDL-C | 2.61 ± 0.72 | 2.61 ± 0.72 | 0.1294 | 2.68 ± 0.73 | 2.69 ± 0.70 | 0.01 | (-0.12, 0.15) | 0.8302 |
